# IS-seq: a novel high throughput survey of in vivo IS*6110* transposition in multiple *Mycobacterium tuberculosis* genomes

**DOI:** 10.1186/1471-2164-13-249

**Published:** 2012-06-15

**Authors:** Alejandro Reyes, Andrea Sandoval, Andrés Cubillos-Ruiz, Katherine E Varley, Ivan Hernández-Neuta, Sofía Samper, Carlos Martín, María Jesús García, Viviana Ritacco, Lucelly López, Jaime Robledo, María Mercedes Zambrano, Robi D Mitra, Patricia Del Portillo

**Affiliations:** 1 Center for Genome Sciences & Systems Biology, Washington University School of Medicine, St. Louis, MO, 63108, USA; 2 Molecular Genetics, Corporación Corpogen, Bogotá, DC, Colombia; 3 Molecular Biotechnology, Corporación Corpogen, Bogotá, DC, Colombia; 4 Hospital Universitario Miguel Servet. IIS Aragón, Zaragoza, Spain; 5 CIBER de Enfermedades Respiratorias (CIBERES), Instituto de Salud Carlos III, Madrid, Spain; 6 Departamento de Microbiología, Medicina Preventiva y Salud Pública, Universidad de Zaragoza, Zaragoza, Spain; 7 Departamento de Medicina Preventiva, Facultad de Medicina, Universidad Autónoma de Madrid, Madrid, Spain; 8 Instituto Nacional de Enfermedades Infecciosas Carlos G Malbrán, Buenos Aires, Argentina; 9 Departamento de epidemiología, Universidad de Antioquia, Medellín, Colombia; 10 Laboratorio de micobacterias, Corporación para Investigaciones Biológicas y, Universidad Pontificia Bolivariana, Medellín, Colombia; 11 Centro Colombiano de Investigación en Tuberculosis (CCITB), Medellín, Colombia

## Abstract

**Background:**

The insertion element IS*6110* is one of the main sources of genomic variability in *Mycobacterium tuberculosis*, the etiological agent of human tuberculosis. Although IS *6110* has been used extensively as an epidemiological marker, the identification of the precise chromosomal insertion sites has been limited by technical challenges. Here, we present IS-seq, a novel method that combines high-throughput sequencing using Illumina technology with efficient combinatorial sample multiplexing to simultaneously probe 519 clinical isolates, identifying almost all the flanking regions of the element in a single experiment.

**Results:**

We identified a total of 6,976 IS*6110* flanking regions on the different isolates. When validated using reference strains, the method had 100% specificity and 98% positive predictive value. The insertions mapped to both coding and non-coding regions, and in some cases interrupted genes thought to be essential for virulence or in vitro growth. Strains were classified into families using insertion sites, and high agreement with previous studies was observed.

**Conclusions:**

This high-throughput IS-seq method, which can also be used to map insertions in other organisms, extends previous surveys of in vivo interrupted loci and provides a baseline for probing the consequences of disruptions in *M. tuberculosis* strains.

## Background

In spite of effective chemotherapy against tuberculosis, this disease is still a global health problem and a leading cause of death worldwide [1]. The causative organism, *Mycobacterium tuberculosis,* is an intracellular pathogen that has infected humans since ancient times [2,3]. Tuberculosis disease (TB) results from an intricate interaction between the host immune system’s efforts to control the infection and the pathogen’s ability to grow and persist within the host. Thus, infection with the tubercle bacillus has variable outcomes that range from sterilizing immunity to active TB [4,5]. Active disease occurs in only 5–10 percent of immunocompetent individuals, with pulmonary tuberculosis being responsible for transmission in the community [6]. In most cases, however, the infection is controlled by the host’s immune system and can lead to the establishment of a long-term latent infection which can reactivate later in life [6].

There is no evidence of recent genetic exchange in *M. tuberculosis* which is thus considered to have a clonal population structure, with strains having almost identical nucleotide sequences [7]. However, there is substantial intra-species genetic diversity that can affect disease outcome [8-11]. The insertion element IS*6110* is an important source of genomic variability for *M. tuberculosis* and is distributed in different positions across the bacterial chromosome [12]. The copy number of this element is highly variable, but most strains contain between 10 and 20 instances. As a result, IS*6110* has been widely used as an epidemiological marker in tuberculosis by DNA fingerprinting using Restriction Fragment Length Polymorphism (RFLP) [13,14]. These insertions also represent “in vivo” transposition events that provide information regarding genes required for human infection and disease. In addition, the persistence of this element in the *M. tuberculosis* genome raises the possibility that it might also drive phenotypic variability or affect strain fitness. It has been demonstrated that IS *6110* transposition can be stimulated by specific stress conditions and thus contribute to genetic diversity in circulating *M. tuberculosis* clinical isolates [15-17]. IS*6110* insertions can affect gene expression by interrupting protein-coding genes, by mediating recombination events that result in deletions and inversions, and by up-regulating the expression of nearby genes due to a promoter located within the transposable element [15,16,18,19]. Thus, although IS*6110* is primarily thought of as a valuable epidemiological tool, its prevalence and effect on genome function prompted us to take a deeper look at the distribution and patterns of IS *6110* insertions by conducting a broad survey of circulating strains.

A previous examination of IS*6110* insertion sites in 161 *M. tuberculosis* isolates, which was accomplished by PCR amplification of the target sequences followed by cloning and sequencing, identified 294 unique insertions sites in only 100 genes, suggesting that a large gene repertoire is required for human infection [20]. However, the technical difficulties and biases due to the amplification and cloning associated with this approach prevented the analysis of a large number of isolates and the detection of all of the insertions present in each strain. To overcome these limitations and assess the extent of naturally occurring IS*6110* insertions during infection and transmission, we used Illumina® sequencing technology to identify IS *6110* flanking regions in more than 500 *M. tuberculosis* isolates from representative collections of clinical isolates from Europe and South America. This high-throughput approach obviates the need for cloning and allows analysis of many strains in a cost-effective manner. We were able to identify approximately 7,000 IS *6110* insertion sites that interrupt almost 300 genes, which together represent <10% of the *M. tuberculosis* genome. Thus, we substantially increased the survey of naturally occurring in vivo IS *6110* transpositions, providing new insights regarding their distribution, diversity and role in strain classification

## Results

### Insertion site sequencing (IS-seq)

**Figure 1:**
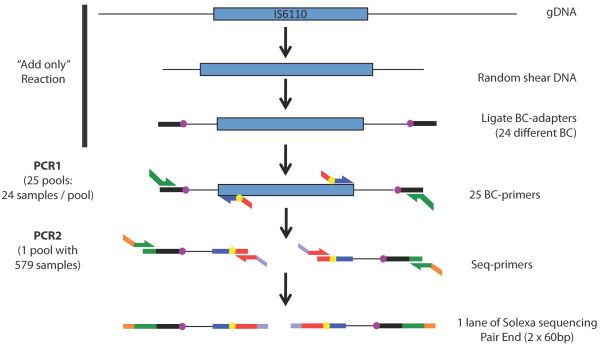
**Overview of IS-seq Method.** Genomic DNA (gDNA) is randomly sheared and ligated to adapters containing 24 different barcodes (BC, purple circles). Amplification from the IS *6110* ends is done in a first PCR (PCR1) with primers that anneal on the IS *6110* (blue) and the adapter (green), incorporating additional BC (yellow circles) and the PE-sequencing primer (red). A second PCR (PCR2) is done to incorporate amplification primers (orange and light blue). Sequencing is carried out using regular PE-primers in an Illumina-GA II.

We developed a method for the simultaneous identification of insertion sites (IS-seq) in up to 600 different strains by barcoding samples and using Illumina® sequencing technology to sequence the genomic DNA flanking both ends of an insertion sequence (Figure 1). We selected 579 *M. tuberculosis* strains for analysis of the IS *6110* insertion sites (see Methods for strain selection criteria). This set included the reference strain *M. tuberculosis* H37Rv (Colombian isolate) and six other strains (3 from Colombia, 2 from Argentina and 1 from Spain) that were used to validate the method by verification of insertion sites using PCR (see Table 1 and Additional file 1: Table S 1). All of the strains included in this study were previously characterized by *IS6110* RFLP and spligotyping.

**Table 1:** Accuracy of the IS-seq method with reference strains

			**Sequence of both sides**				**Sequence by a single termini**			
Strain	RFLP bands	PCR confirmed	#IS sites	False Positives	False Negatives	True Positives	#IS sites	False Positives	False Negatives	True Positives
Col-H37Rv	13	15	15	0	0	15	17	2	0	15
Col-UT98	11	13	10	0	3	10	13	0	0	13
Col-UT98C	11	13	10	0	3	10	13	0	0	13
Col-UT261	10	14	13	0	1	13	14	0	0	14
Arg-410	9	12	9	0	3	9	12	0	0	12
Arg-6548	8	11	8	0	3	8	11	0	0	11
Spa-C1	10	15	11	0	4	11	15	0	0	15
Total	72	93		0	17	76		2	0	93
Sensitivity	77%					82%				100%
PPV*					100%					98%

*PPV: Positive Predicted value.

For each reference strain the number of RFLP observed bands is reported as well as the number of IS sites identified using IS-seq by one or both termini of the insertion and the number of confirmed sites by PCR. Sensitivity and Positive Predicted value is calculated as indicated in Methods.

To generate a sequencing library we randomly sheared genomic DNA and ligated barcoded adapters to the fragment ends. Fragments that include the terminal end of the IS*6110* were then amplified in a nested PCR using primers that incorporate additional barcodes. Finally, the amplified products were pooled, purified and sequenced. The resulting fragments contain either the 3’ or 5’ terminal sequence of IS *6110*, the flanking genomic sequence, and two different barcodes at each terminus. Since most of the enzymatic steps are “add only” reactions, there is no material loss due to purification steps and the protocol can be easily automated and performed in 96-well plates. Furthermore, reactions are pooled after both barcodes have been added, so that the gel purification of hundreds of libraries can be performed using a single gel lane.

Using this protocol, we obtained more than 14 million “paired-end” sequences using an Illumina® GA II machine. The sequence reads contained an even distribution of barcodes (Additional file 2: Figure S 1), showing that pooling before the gel extraction without individual quantification introduced no bias on the distribution. We were able to identify sequences for all of the 579 strains with an average of 7,042 reads per strain. To increase the precision of mapping, the short reads were sorted by sample and assembled to generate contigs of up to 300 nucleotides before being mapped to the reference *M. tuberculosis* genome. All assembled contigs were mapped to at least one of the available genomes. The sequences that did not assemble in contigs were further mapped as single reads to the different genomes and less than 5% of these reads could not be mapped. Based on an estimate of the average sequence coverage obtained per IS *6110* element (i.e. number of reads per RFLP band), a cutoff point was defined (see Methods) to remove strains with low coverage, which are likely to produce unreliable results (Additional file 3: Figure S 2). After this filtering, 519 strains were retained for analysis (Additional file 1: Table S 1). We included a negative control species in this study that lacks IS*6110* ( *Mycobacterium avium*) and obtained only few and scattered reads without significant mapping. These results demonstrate the efficiency and specificity of the protocol and primer design.

### Accuracy of the method

The accuracy of IS-seq was assessed using seven *M. tuberculosis* strains for which 72 insertions had been previously characterized by RFLP fingerprinting. IS-Seq identified these 72, as well as 23 additional insertions. Of these additional insertions, all but two were confirmed by PCR (Table 1). These two false positives corresponded to two insertions in the H37Rv reference strain (Col-H37Rv) that were mapped to only one terminus of the IS*6110* element. Thus, by requiring sequence from both ends of the IS *6110* element 100% specificity could be achieved, at the cost of sensitivity (Table 1). The identification of additional insertion sites using IS-seq was not altogether surprising given the limited resolution of the RFLP fingerprinting method. Based on the analysis of these strains, the IS-seq method had a sensitivity of 100% and a positive predictive value of 98% (Table 1). It was also interesting to find that while the Col-H37Rv strain used here had 15 copies of IS*6110*, the sequenced genome of *M. tuberculosis* H37Rv (Accession number NC_000962.2) had 16 IS *6110* elements, with an extra copy being part of a doublet in positions 3,552,584 and 3,552,713 [21], highlighting differences in the genetic makeup of these reference strains. The reproducibility of the method was assessed by including two sets of duplicate samples of the same DNA (Arg14015-Arg1545 and Spa32-SpaC1); the agreement on the number and insertion position within each pair of DNAs was greater than 90% (Additional file 1: Table S 1).

A hallmark of the IS*6110* element insertion is the generation of a 3-4 bp duplication at the insertion site [22]. We expected to identify this duplication in those cases where we were able to map the insertion site of the IS*6110* element on both termini (73.1%). In 91.85% of these cases we identified a 3 bp (75.56%) or 4 bp (16.29%) duplication. Taken together, these results demonstrate that the IS-seq method accurately and reproducibly identifies and maps IS *6110* insertion sites.

### Genomic distribution of the IS*6110* element

**Figure 2:**
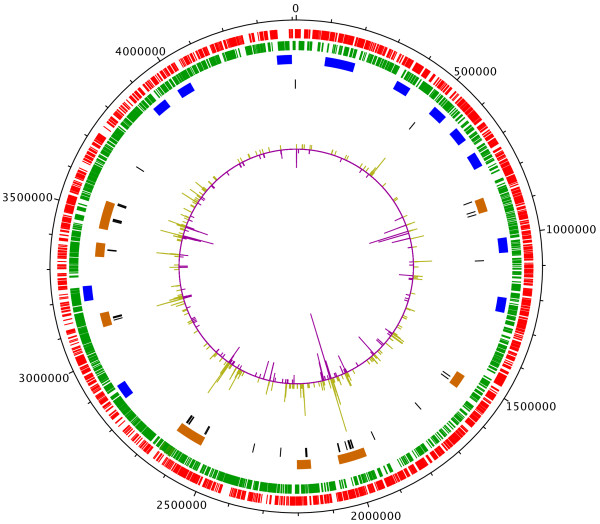
**Genomic Distribution of IS*6110*Elements.** The *M. tuberculosis* H37Rv circular genome is shown with outermost circles representing genes located on the positive (red) and negative (green) strands. Inner circle shows insertions in non-coding (purple) and coding (yellow) regions, with peak sizes scaled to indicate number of unique insertions per 200 bp window. Black lines indicate 2 kb regions with significantly more insertions than expected by a uniform distribution, brown boxes are 50Kb regions with significantly more insertions and blue boxes indicate regions of 50Kb with significantly less insertions.

The IS-seq method identified 6,976 insertion sites in the 519 strains analyzed (Additional file 1: Table S 1). These represented 964 different insertions that affected 433 loci (Additional file 1: Table S 2), 66% of which were in coding regions (286 ORFs) and 34% were intergenic (147). This represents a significant bias towards non-coding regions, since intergenic regions account for only ~10% of the *M. tuberculosis* genome. An analysis of the distribution and frequency of independent insertion sites throughout the genome also showed a significant location bias. A Poisson test for the distribution of insertion sites showed that there were forty-four 2 Kb-windows (~2.5% of the genome) with significantly more insertions than expected (Figure 2 and Additional file 1: Tables S 3a, b and c). When 50 kb windows were used, regions with significantly more or less insertions were also identified (Figure 2). An analysis of these regions revealed no significant correlation with GC content (R^2^: 0.03; p value 0.1549).

We found multiple insertion sites in 40% of the affected loci (Additional file 1: Table S 4). Of these loci, 17 contained 6 or more independent sites and thus were labeled as “hotspots”, with the “hottest” locus containing 23 different insertion elements (Additional file 1: Table S 4). Several of these “hotspots” have been previously described, such as *mmpL12* (Rv1522c), which is thought to be involved in fatty acid transport, or *plcD*, which encodes a phospholipase C, and intergenic regions like *dnaA-dnaN* or the region between genes Rv2813-2816 (Additional file 1: Table S 2). This latter locus corresponds to a Cluster of Regularly Interspaced Short Palindromic Repeats (CRISPR) known as the Direct Repeat (DR) region, which is the template for the spoligotyping technique [23]. We identified 577 insertions in 16 different sites within the DR region (Additional file 1: Table S 2), one of which was present in 485 (93.4%) of the evaluated strains, including the reference H37Rv genome (genes Rv2814c-Rv2815c located between DR spacers 24 and 25). Interestingly, this is the only IS*6110* present in the vast majority of *Mycobacterium bovis* isolates, suggesting that it could have been present before many of the modern lineages diverged from each other. We therefore refer to this as an ancestral point of insertion. This insertion was absent in the Beijing strains evaluated in our study, which is congruent with the loss of spacers 1–34 in the DR region that characterizes this *M. tuberculosis* family [24].

To test if our analysis was close to saturation in terms of identifying all possible genes that could be naturally interrupted by the IS*6110* element, a rarefaction analysis was carried out (Additional file 4: Figure S 3). The lack of a plateau indicating saturation in mutable sites shows that additional insertions in coding sequences could still be identified in circulating *M. tuberculosis* strains. This study was done with representatives of the major lineages circulating in Europe and Latin America and falls short of representatives of strains prevalent in Africa and Asia that may harbor distinct insertion sites. A regression analysis on the rarefaction curves estimated that between 278 and 371 genes could support insertion sites, consistent with previous estimates of the number of possible in vivo mutations [20]. The presence of mutable sites results from an ongoing evolutionary process that in *M. tuberculosis* might not necessarily have reached saturation. Thus, even if we sampled all available isolates there is no guarantee that we could observe such saturation of in vivo insertions.

### Analysis of disrupted genes

We next characterized the genes disrupted by IS*6110* insertions by assigning them to functional categories based on the NCBI Clusters of Orthologous Groups (COGs) (http://www.ncbi.nlm.nih.gov/sutils/coxik.cgi?gi=135) and the Tuberculist database (http://tuberculist.epfl.ch/index.html). Disrupted genes were broadly distributed across functional categories, with a significant over-representation of insertions in genes belonging to three categories: 1) PE/PPE, 2) genes in COG from the category “unknown” and 3) Cell wall/membrane/envelope biogenesis genes (Table 2). The PE and PPE gene families encode glycine-rich proteins that contain repeats of the motifs Pro-Glu (PE) or Pro-Pro-Glu (PPE) and represent about 10% of the predicted proteins in *M. tuberculosis*[21]. Within the PE/PPE gene family, the majority of interrupted loci (23) occurred in PPE genes. Under-represented COG categories included genes involved in lipid and amino acid transport and metabolism, energy production and conversion, as well as the category defined as virulence, detoxification and adaptation in Tuberculist (Table 2).

**Table 2:** Functional categories of interrupted genes

				**Fisher exact test**^**5**^	
**Code**	**Category**	**Cumulative**	**Independent**	**p-value**	**p-value**
		**Length**^**3**^	**Insertions**^**4**^	**(Over-represented)**	**(Under-represented)**
	**PE/PPE**^**1**^	280.083	96	3.97E-16	1.00E + 00
-	Not in COG	670.659	158	1.12E-11	1.00E + 00
M	**Cell wall/membrane/envelope biogenesis**	144.558	36	4.76E-04	1.00E + 00
T	Signal transduction mechanisms	119.979	24	3.72E-02	9.63E-01
A	RNA processing and modification	648	0	8.75E-02	9.13E-01
N	Cell Motility	657	0	8.87E-02	9.11E-01
L	Replication. recombination and repair^2^	213.870	37	8,98E-02	9.10E-01
K	Transcription	175.641	24	5,13E-01	4.87E-01
V	Defense mechanisms	46.863	5	6,50E-01	3.50E-01
R	General function prediction only	449.865	57	7.88E-01	2.12E-01
S	**Function unknown**	199.185	23	8.14E-01	1.86E-01
H	Coenzyme transport and metabolism	171.450	19	8.37E-01	1.63E-01
D	Cell cycle control, cell division. chromosome partitioning	52.116	3	9,37E-01	6.35E-02
F	Nucleotide transport and metabolism	70.152	4	9.70E-01	3.00E-02
U	Intracellular trafficking, secretion. and vesicular transport	24.903	0	9.71E-01	2.93E-02
O	Posttranslational modification, protein turnover. chaperones	114.210	7	9.92E-01	8.51E-03
Q	Secondary metabolites biosynthesis. transport and catabolism	341.379	28	9.99E-01	7.55E-04
I	**Lipid transport and metabolism**	289.923	19	1.00E + 00	6.52E-05
J	Translation. ribosomal structure and biogenesis	138.906	4	1.00E + 00	1.83E-05
G	Carbohydrate transport and metabolism	168.417	6	1.00E + 00	1.15E-05
P	Inorganic ion transport and metabolism	159.159	4	1.00E + 00	1.61E-06
C	**Energy production and conversion**	258.336	10	1.00E + 00	1.15E-07
E	Amino acid transport and metabolism	247.980	4	1.00E + 00	1.76E-11
	**Virulence. detoxification and adaptation**^**6**^	147.877	7	1.00E + 00	3.55E-04

1. Proteins belonging to the PE/PPE families were extracted to a separate category, as they constitute an important family of proteins in *M. tuberculosis.*

2. The genes for the transposase IS*6110* were removed from the category of Replication, recombination and repair.

3. Cumulative length (bp) of all the genes in a given category. The probability of under or over-representation of a given functional category is dependent on both the number of genes and their length.

4. Represents the number of independent insertion events identified in genes of a given category.

5. Probability of over or under-representation of insertion sequences interrupting genes of a given category. In bold, categories with significant over-, under-representation after Bonferroni correction. Bonferroni corrected threshold = 2.2E-3.

6. This category is not part of COG; it is defined in tuberculist (see Methods).

We next compared the set of ORFs harboring an IS*6110* element with previous studies that identified *M. tuberculosis* genes required for growth in vitro or in vivo [25-30]. Twenty-seven genes previously identified as being essential under different experimental conditions were found to contain IS*6110* elements (Table 3). Seven of these genes contained the insertion element in the terminal 20% of the gene, which although less likely to affect function could still do so in proteins with C-terminal active sites or domains. To rule out the possibility that these observations were due to an artifact of the IS-Seq method, we validated these insertion sites by PCR. Twenty-four of the 27 insertions were confirmed (Table 2), and one locus failed to amplify, probably due to the limited amount of DNA used as starting material (See Methods).

**Table 3:** Insertions in genes important for growth or virulence

**Locus**	**Gene ID**	**Description**	**IS position within ORF (%)**^**1**^	**PCR verification**^**2**^	**Reference**
**Essential genes**					
Rv0336	-	Conserved 13E12 repeat family protein	71.2	+	[25]
Rv0405	pks6	Probable membrane bound polyketide synthase	26.7	+	[26]
Rv1371	-	Probable conserved membrane protein	17.8	+	[29]
Rv1469	ctpD	Probable cation transporter P-type ATPase D	2.5	+	[29]
Rv1477	ripA	Peptidoglycan hydrolase	72.9	+	[28,30]
Rv1753c	PPE24	PPE Family protein	2.4	+	[26]^,^[25][27]
Rv1978	-	Conserved hypothetical protein	41.8	+	[27]
Rv2388c	hemN	Coproporphyrinogen III oxidase	28.7	-	[29]^,^[27]
Rv2708c	-	Conserved hypothetical protein	61.8	+	[27]
Rv2808	-	Hypothetical protein	23.6	+	[29]
Rv2812	-	Probable transposase	8.8	+	[28,30]
Rv2817c	-	Conserved hypothetical protein	71.8	+	[28,30]
Rv3018c	PPE46	PPE family protein	4.7	+	[28,30]
Rv3112	moaD1	Probable molybdenum cofactor biosynthesis protein D	29.8	+	[28,30]
Rv3113	-	Possible phosphatase	34.1	+	[28,30]
Rv3114	-	Conserved hypothetical protein	40.7	-	[29]
Rv3201c	-	Probable ATP-dependent DNA helicase	0.2	+	[28,30]
Rv3229c	desA3	Possible linoleoyl-CoA desaturase	43.4	+	[29]^,^[27]
Rv3343c	PPE54	PPE family protein	9.3	+	[28,30]
Rv3376	-	Conserved hypothetical protein	60.9	+	[27]
**Essential genes - Less probable to affect function**					
Rv0001	dnaA	Chromosomal replication initiation protein	95.1	+	[28,30]
Rv0755c	PPE12	PPE Family protein	96.4	+	[28,30]
Rv2282c	-	Probable transcription regulator (LysR family)	84.9	+	[27]
Rv2833c	ugpB	Probable Sn-glycerol-3-phosphate-binding lipoprotein	88.9	+	[28,30]
Rv2856	nicT	Possible nickel-transport integral membrane protein	89.8	+	[28,30]
Rv3111	moaC1	Molybdenum cofactor biosynthesis protein C	92.8	-	[28,30]
Rv3177	-	Possible peroxidase	90.4	+	[28,30]
**Virulence, detoxification and adaptation category (Tuberculist)**					
Rv0591	mce2C	MCE-family protein	56.1		
Rv1477	ripA	Peptidoglycan hydrolase	72.9		
Rv1720c	vapC12	Possible toxin	7.9		
Rv2494	-	Conserved hypothetical protein	97.2		
Rv3176c	mesT	Probable epoxide hydrolase	11.8		
Rv3177	-	Possible peroxidase	90.4		
Rv3473c (MT3579	bpoA	Possible peroxidase	93.8		

1) Indicates the position of the insertion within the ORF as the percentage of gene upstream of the insertion.

2) Insertions verified using PCR: +, PCR positive; -, PCR negative.

### IS*6110* family signatures

**Figure 3:**
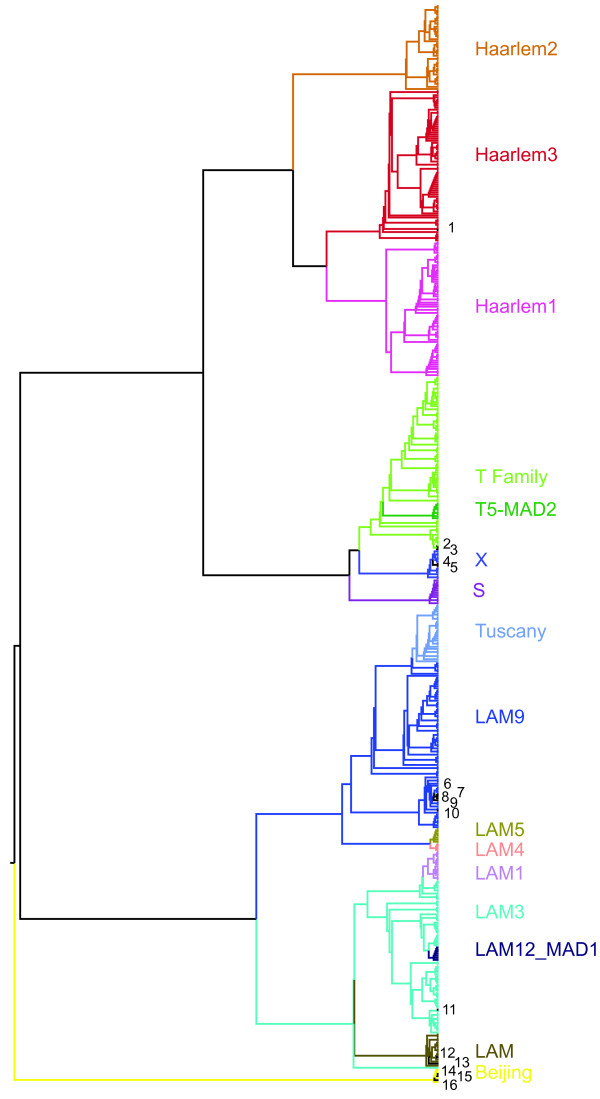
**Classification of*M. tuberculosis*Strains Based on IS*6110*Insertions.** An UPGMA tree was generated from the distance matrix obtained using Variation of Information metric for 504 strains. Colors represent the main lineage 4 families present in the study, using Beijing strains (lineage 2) as an outgroup. Reference sequenced bacterial genomes are indicated by numbers on the side: 1- TB str Haarlem, 2- TB H37Rv, 3- TB H37Ra, 4- TB CDC-1551, 5-TB-C, 6- TB KZN_4207, 7- TB_KZN_R596, 8- TB_KZN_V2475, 9- TB_KZN_1435, 10- TB_KZN_605, 11- TB_F11, 12- TB GM_1503, 13- TB 98-R604, 14- TB T85, 15- TB 210, 16- TB 02_1987 (see Methods).

To see how well these IS*6110* patterns correlated with known strain classifications, we examined the relationship between IS *6110* distribution and *M. tuberculosis* strain families (as determined by spoligotyping [24]). The use of specific IS*6110* insertions as markers for different *M. tuberculosis* lineages has been described. For example, the Beijing lineages have been described to contain an IS *6110* insertion in the intergenic region between *dnaAdnaN*[31] and the spoligotype characteristic of the Haarlem lineage (spoligotype 50) is partially due to a characteristic IS*6110* in the DR region [32]. Despite the limitation of using the IS*6110* to accurately discriminate between the different *M. tuberculosis* phylogenetic lineages [33], we propose that the precise locations of IS*6110* insertions could be useful as markers within a lineage and, furthermore, could help to identify previously uncharacterized clades within families. We therefore used a Mutual Information metric (see Methods) to identify the co-occurrence or absence of insertion sites in order to generate a dendrogram using 504 of our strains, which belonged to the *M. tuberculosis* Euro-American lineage (or Lineage 4; 500 strains) and the Beijing family (Lineage 2; 4 strains), which was also used as an outgroup (Figure 3). We also included the 16 publicly available genomes that belong to either Lineage 2 or 4 (see Methods). The tree generated distinguished the main Lineage 4 families (LAM, Haarlem, T, S, X), and subdivisions within families, such as the H1, H2 and H3 Haarlem sub-families defined by spoligotyping. For the LAM family, two main clusters were formed, one composed of LAM3, LAM1, LAM12 and the second of LAM9, LAM4, LAM5 and Tuscany. The T family also showed sub-clusters that did not reflect the T sub-families described by spoligotyping, but were consistent with findings using SNPs for classification that suggested that T sub-families are still not well defined [34].

By analyzing the patterns of insertion events in the different families, we identified 138 family-specific insertions, consisting of few insertions per family (Additional file 1: Table S 5). These signature IS sites helped to understand inconsistencies in spoligotyping (Additional file 1: Table S 6). In particular, 20 strains ambiguously classified by spoligotyping as either ‘Haarlem orphan, H1 and H3, and only as Haarlem by RFLP, were found to contain a H3 subfamily characteristic insertion site and a H1 subfamily spoligotype pattern in the DR region, which may have occurred independently as a product of recombination between two IS*6110*. Interestingly, we were able to identify the insertions in the DR region that are responsible for the spoligotype patterns of the different members of the Haarlem family (Additional file 1: Table S 5). Two Haarlem 4 strains were found to be Ural strains, consistent with recent reports [34,35], and the Tuscany subfamily was closely related to the LAM subfamily rather than the T family (Figure 3), as had been recently suggested [34]. Also, the previously described insertion in the *dna*A *-dna*N intergenic region in Beijing isolates was identified in all 4 strains. When these family-specific insertions were used to classify the 519 strains used in this study, family or subfamily information was provided for 513 strains compared to 500 that were classified by spoligotyping. These signatures were also used to classify 16 of the 23 available complete *M. tuberculosis* genomes (see Methods) (Additional file 1: Table S 7, Figure 3). Some strains were assigned to specific families, as was the case of the reference H37Rv and the H37Ra strains that were assigned to the T family, and the F11 and Haarlem strains that were identified as belonging to LAM3 and Haarlem 3, respectively. Other strains were classified as LAM9 (KZN 1435, KZN 4207, KZN 605, KZN R596, KZN V2475), LAM ('98-R604 INH-RIF-EM' and GM 1503), X (CDC-1551, TB-C) or Beijing (210, 02_1987, T85); all in 100% agreement with previously suggested families, when available (see Methods) [33]. The inability to classify the remaining 7 cases is due to the fact that they belong to lineages not significantly represented in our study (Additional file 1: Table S 7). These results show that classification of tuberculosis strains by the use of signature IS sites is consistent with SNP classification and therefore highly reliable.

To address the possibility that insertions could appear independently in different lineages because they confer some selective advantage, such as drug resistance, we performed a Conditional Mutual Information analysis [36] to see if any insertion sites correlated significantly with the epidemiological data collected for each strain (patient gender, age, country of isolation, HIV status, acid-fast bacilli smear result, strain drug resistance profile and transmissibility, this latter assessed as size of IS*6110* RFLP cluster). No association was found between insertion sites present in different phylogenetic families and a specific biological trait, suggesting that it is unlikely that exact, yet independent insertions could occur in different families that confer a fitness advantage (Additional file 1: Table S 8). Furthermore, in all cases where a specific insertion sequence was present in multiple strains, the strains containing that insertion belonged to the same phylogenetic lineage, suggesting that occurrence of independent insertions in the same nucleotide position but in different lineages by convergent evolution, although plausible, is very unlikely. Instead, hotspots of insertions occurring in a single locus within few nucleotides of distance were observed (see above).

## Discussion

The IS-seq method was effective at detecting, in a single reaction, multiple IS*6110* insertions sites in over 500 *M. tuberculosis* isolates from diverse geographical origins and phylogenetic lineages. This was achieved using a novel approach with the following features: i) the use of two molecular barcodes per sample to multiplex samples on an Illumina® sequencer, allowing the analysis of a combinatorial number of samples (600) with just 25 different barcodes; ii) a high-throughput library construction protocol that uses minimal amounts of starting material, and iii) a novel computational strategy that maximizes the efficiency of sequence identification by assembling reads prior to mapping against the *M. tuberculosis* sequenced genomes. This study, combined with the advances on sequencing technologies, provides a highly cost-efficient method for *M. tuberculosis* strain identification and typing that could be extended to other target amplifications and sequencing. While the use of 4 bp barcodes allows multiplexing of up to 600 samples per sequencing reaction, 5 bp barcodes may allow pooling up to 9,216 samples. Additionally, the requirement of just 100 reads per insertion site for accurately mapping, combined with new technologies such as MiSeq that achieve up to 4 million reads in just a matter of hours, can revolutionize the field of tuberculosis strain characterization.

IS-seq effectively detected loci with multiple independent insertions or insertions in the same location in many strains, and identified new and previously characterized hotspots and insertions that could be considered ancestral. Although an IS*6110* element present in the same locus could be due to convergent evolution, which in time could be reflected as insertion hot spots (i.e. independent insertions within few nucleotides in a given locus)[10], our observations suggest that insertions at the same nucleotide position are more likely due to a common ancestral insertion. Strategies similar to IS-seq have been recently developed for identifying microbial transposon insertions sites generated during in vitro mutagenesis [37,38] and endogenous transposon elements in the human genome [39-41]. However, our method differs because it can identify multiple insertions sites per strain and simultaneously analyze hundreds of different strains per reaction. The advantage of IS-seq results from a more streamlined library preparation protocol and a more efficient barcoding strategy.

The insertion sites identified in these 519 clinical *M. tuberculosis* isolates significantly extends previous surveys by identifying more that 900 new sites and provides, for the first time, a comprehensive overview of the gene repertoire that can be interrupted in vivo by IS *6110.* Rarefaction curves indicated that additional genes could still be identified, especially in strains that were not significantly represented in our dataset. However, the number of loci predicted to sustain in vivo insertions was close to the maximum of about 300 genes previously predicted based on an analysis of 161 strains [20]. It is also consistent with the observation that the number of naturally occurring in vivo mutations is much lower than the genes predicted to be non-essential using transposon mutagenesis approaches [20], probably due to the detrimental effect of cumulative insertions on in vivo fitness. The smaller gene set that can be inactivated in vivo when compared to in vitro studies, tied to identification of insertions in previously predicted essential genes, indicates evident differences in the conditions imposed on circulating strains as they adapt to the human host. In this respect, the overrepresentation of insertions in PE/PPE, cell wall and membrane biogenesis genes and ORFs of “unknown function” could reflect adaptation as bacteria alter envelope components in response to pressure imposed by the host environment. In particular, the presence of IS*6110* elements in the PE/PPE gene family reinforces their previously proposed role in immune evasion and is consistent with previous reports [20,42,43]. However, the over-representation of certain functional categories could also be due to redundancy in function that might alleviate the effect of gene disruption. In contrast, underrepresented gene categories, such as energy production and conversion, highlight their importance in processes required for bacterial virulence and survival. Finally, the identification of IS*6110* insertions in genes reported to be essential is not implausible, since insertions as well as deletions of some of these genes have already been described [20,44]. In addition to gene disruption, the IS*6110* insertions identified here can also result in transcription up-regulation of downstream genes, as has been reported for an insertion in the promoter region of the two-component system gene *phoP*[18]. A similar insertion in the *phoP* promoter was observed here in a fast evolving clade of Haarlem 1 in Latin America (Additional file 1: Table S 5). This survey therefore broadens the list and emphasizes again the difference in requirements between strains grown and studied under laboratory conditions and those subjected to the pressure of in vivo passage and processes of natural infection in the human host.

Interestingly, the strain classification based on the distribution of IS*6110* using IS-seq was congruent with that based on spoligotyping and SNPs for sequenced genomes. Both *IS6110* RFLP and spligotyping have been widely used for epidemiological studies but were considered to have limited value as phylogenetic tools [33]. Very recently, however, the accuracy of spoligotyping for sublineage classification has been acknowledged [45] and IS*6110* has been shown to yield high phylogenetic resolution when used as target in a fluorescent amplified fragment length polymorphism technique [46]. Similarly, the IS-seq strategy presented here resolves precise insertion site patterns characteristic of various strain families, thus providing robust phylogenetic information. It remains to be seen, however, if the signatures identified herein will remain family-specific as more strains are analyzed from lineages not included in the present study. Although no correlation was found between IS*6110* insertions present in different families and particular biological traits, we cannot rule out the possibility that clade specific insertions can alter important biological features of a strain and hence contribute to the spread of a new clade. This study can serve as basis for additional analyses regarding the possible effects of these elements in clinical isolates and whether they may influence clonal spread by playing a role in strain fitness.

Thus, this study presents a new method for efficiently typing naturally occurring insertion sequences in hundreds of isolates simultaneously. It also expands the current inventory of in vivo interrupted loci, gives new insights into the genetic requirements in circulating strains and opens the way to understanding the consequences of insertions and the role of IS*6110* in the biology and pathogenesis of *M. tuberculosis*. Furthermore, this approach can be used to study the natural history of insertion elements and transposons in the genomes of other pathogenic organisms where limited DNA quantity and complex genome structure have made this pursuit challenging. As costs of DNA sequencing decrease, sequenced-based approaches will probably become more attractive for strain typing in a near future. However, in a bacterial genome as stable as *M. tuberculosis* an approach that specifically targets variable regions, such as insertion sites that can provide sufficient phylogenetic information, may still prove more tractable than full genome re-sequencing of strains.

## Conclusions

This article describes the simultaneous identification of IS*6110* flanking regions in a large collection (more than 500) of *M. tuberculosis* clinical isolates. This is an innovative approach that combines an efficient barcoding strategy with a 96-well plate automated library preparation and massive parallel sequencing in a single Illumina sequencing reaction. Our results showed about 7,000 different insertions in the whole panel of analyzed isolates, which is more than has been identified up to date. Among the interrupted genes we identified some that were proposed to be essential for virulence and in vitro growth. These results will be useful for researchers in the field of tuberculosis including the development of new drugs and vaccines.

## Methods

### Selection of isolates

The strains for this study were selected from three different collections in Colombia, Argentina and Spain and were isolated from patients between 1995 and 2008. Isolates were selected to represent the main circulating lineages as well as strains with particular characteristics, such as strains involved in TB epidemics, or strains that acquired multidrug resistance. All isolates were typed by spoligotyping and IS*6110* RFLP, as described [23] and were selected based on diverse RFLP patterns. Spoligotypes were assigned by comparison with the international spoligotype database, SpolDB4 (http://www.pasteurguadeloupe.fr:8081/SITVITDemo/index.jsp) [24].

### Ethics statement

The study and protocols for collection of bacterial strains from patients were approved by the ethics committees at each institution: the Ethics Committee at the Universidad de Antioquia and local health authorities in Medellín, Colombia (Dirección Seccional de Salud de Antioquia and the Secretaría de Salud de Medellín), the Ethical Committee of the Aragon Government for strains collected at the Hospital of Getafe, Hospital Universitario Miguel Servet and Hospital Universitario Lozano Blesa in Spain, and the Ethical Committee of the Instituto Malbrán in Argentina. Written informed consent was provided by the subjects in Colombia and Spain. In Argentina this was waived because the investigators performing the study received secondary data about human subjects and microbiological strains, which had been originally isolated elsewhere from human biological samples for diagnostic purposes, but no possible personal identifiers were transferred to the researchers. The source of the data was disclosed to the Committee in the application.

### DNA isolation

Extraction of genomic DNA was performed by the chemical lysis method [47]. In brief, all visible colonies grown on Lowenstein-Jensen were collected into 400 μl 10 mM Tris- 1 mM EDTA buffer pH 8.0, suspensions were heated at 80°C for 20 min and incubated with lysozyme at 37°C overnight. Cells were then incubated with SDS and proteinase K (10% SDS and 0.05 mg/mL proteinase K) at 65°C for 10 min, 100 μl of a CTAB-NaCl solution (10% CTAB, 0.7 M NaCl) were added, tubes were vortexed until the suspension turned milky, and incubated for 10 min at 65°C. DNA was purified and precipitated using chloroform:isoamylalcohol (24:1) and 0.6 volumes of isopropanol, respectively. The genomic DNA was then resuspended in 20 μl of nuclease-free water.

### Preparation of DNA libraries

The DNA from each sample was quantified by spectroscopy (NanoDrop®, ThermoScientific). Four hundred nanograms of each DNA were diluted in 10 μl of sterile water and sonicated at maximum power for 1 min (XL 2020 Sonicator, Misonix) to yield fragments with an average size between 200–400 bp. End repair of the DNA was done in a final volume of 20 μl using 10 μl of sonicated DNA, 0.05 mM dNTPs, 1x T4 DNA ligase buffer (NEB), 5 U of Klenow DNA Polymerase (NEB) and 10 U of T4 PNK (NEB). The reaction was incubated for 30 min at 25°C followed by 20 min at 75°C. To remove unused dNTPs, 11U of SAP (Promega) were added to each sample and incubated 30 min at 37°C followed by 30 min at 80°C to inactivate the phosphatase. Addition of a 3’ terminus adenine to the fragments was done in 30 μl using 21 μl of the previous reaction, 0.1 mM dATP, 10 U of Klenow exo minus (Epicentre) and 3 μl of 1x T4 DNA Ligase buffer; the reaction was incubated for 30 min at 37°C and then 20 min at 75°C.

Adapters were annealed by combining 25 picomoles of the forward and reverse adapters (Adapter F: P –B’AGATCGGAAGAGCGGTTCAGCAGGAATGCCGAG and Adapter R: ACACTCTTTCCCTACACGACGCTCTTCCGATCTBT [Illumina; Oligonucleotide sequences © 2007–2009 Illumina, Inc. All rights reserved], where B indicates the barcode position (Additional file 1: Table S 9) and B’ the reverse complement, P designates a phosphate) in 2.5X T4 DNA ligase buffer in a final volume of 20 μl. The solution was heated for 2 min at 94°C and slowly cooled down (0.1°C/s) to room temperature. The adapter mix was added to the previously prepared genomic DNA (30 μl) in a 50 μl reaction volume and 400 U of T4 DNA ligase (NEB) were added and incubated for 1 hour at 16°C followed by 10 min at 65°C. QiAquick PCR purification kit (Qiagen) was used to clean ligated DNA, which was eluted in 30 μl of Elution Buffer (EB).

### PCR amplification of insertion sequence flanking sites

For the amplification of regions flanking the IS*6110* element, sets of 24 samples that were prepared and ligated with different barcoded adapters were pooled. Twenty five different pools were amplified, each using a IS *6110*-specific primer tagged with a 4–5 bp barcode (Additional file 1: Table S 9) and the 3’ end of the Pair End (PE) adapter (Illumina®) (IS-right: 5’ CTGAACCGCTCTTCCGATCT**B**ACTCACCGGGGCGGTT 3’; IS-left: 5’ CTGAACCGCTCTTCCGATCT**B**ACATGCCGGGGCGGTT 3’, where B indicates the barcode and adapter sequences are underlined), and Nested_Primer1 that anneals on the adapter (underlined) and contains the reverse Illumina PE PCR primer 1.0 (italics) (5’ *AATGATACGGCGA*CCACCGAGATCTACATCTTTCCCTACACGAC 3’). IS-specific primers were designed to specifically anneal the terminal direct repeats of the IS *6110* leaving a CA dinucleotide at the 3’ end for quality control. The PCR was performed using 5 U of Platinum Taq (Invitrogen), 1x PCR Buffer, 0.5 mM dNTPs, 0.5 μM of Adapter Primer1and 0.5 μM of an equimolar mix of the IS specific primers, 2 mM MgCl_2_, 0.5 M Betaine and 120 ng (equivalent to 5 ng per sample) of the purified DNA in a final volume of 25 μl. The thermal cycling program consisted of 33 min at 93°C for initial denaturation and 35 cycles of 2-step PCR amplification using 93°C for 45 sec and 6 min annealing/amplification. The first 5 cycles were done at 55°C, the remaining were done at 68°C.

Nested_Primer1 was then used in a nested PCR reaction with Nested_Primer2 (5’ *CAAGCAGAAGACGGCATACGA*GAT CGGTCTCGGCATTCCTGCTGAACCGCT3’) that anneals on the adapter (underlined) and incorporates the Illumina PE PCR Primer 2.0 (italics). This was done using 2 μl of the initial product; all the reagents had the same concentration as the initial PCR. The amplification was done as before for 20 cycles with an annealing temperature of 62°C. From this PCR, 2.4 μg of DNA from each reaction (100 ng/sample) were pooled together in a single tube. Purification of the fragments between 200 – 500 bp was done by gel extraction (QIAquick, Qiagen) and the purified DNA was sequenced in an Illumina® Genome Analyzer (GA) II sequencer. Two lanes in independent flow cells were performed. The first one generating 2 x 35 bp PE sequences and the second one generating 2 x 60 bp PE sequences. Generated sequences were deposited in NCBI Short Read Archive accession number SRA030755.

### Method validation using reference strains

Seven strains (three from Colombia, two from Argentina, one from Spain and the reference *M. tuberculosis* H37Rv (Colombian Isolate) with known insertion sites were used to validate the method. Primers were designed to PCR-amplify genes and regions flanking the insertion sequence (Additional file 1: Table S 10) such that a difference in size of the amplified product reflects the presence of the insertion element (1362 bp). Twenty nanograms of genomic DNA were used for the PCR in a final volume of 25 μl with, 1X Red Mix (Corpogen, Colombia), 400 nM of each primer and purified water. Three temperature profiles were established according to the expected size of the amplified product. For products of more than 2500 bp, 95°C for 5 minutes followed by 40 cycles of 95°C for 45 sec, annealing temperature depending on primer sequence for 45 sec and 72°C for 2.5 min, and final extension at 72°C for 10 min. For products between 1500 and 2400 bp, initial denaturation at 95°C for 5 min, 35 cycles of 95°C for 40 sec, annealing temperature for 40 sec and 72°C for 2 min with final extension for 10 min at 72°C. For small products, the number of cycles was reduced to 30, denaturation and annealing time reduced to 35 sec and extension to 1 min, initial and final extension remained the same in all three profiles. Amplified products were visualized in 1% agarose gel stained with ethidium bromide.

### Sequence data processing

Sequences were processed using a combination of stand-alone software linked through perl scripts for the parallel analysis of all the sequences. The standard sequencer output consists of two files, one for each pair-end, with the corresponding sequences in the same position on each file. In our particular case, one file had sequences composed by the adapter barcode followed by the target sequence (referred to as “adapter read” hereafter), the other file has sequences containing the primer barcode, the IS-Primer, a CA dinucleotide characteristic of the insertion site and the target sequence (called a “primer read” hereafter).

The reads were first classified according to the barcodes, filtered for low quality data and the IS primer was masked. Trimming of possible sequencing primers was done using agrep (http://laurikari.net/tre/) for fuzzy string matching with up to 2 errors in the IS primer and posterior masking. A Smith-Waterman fast alignment was then used to search for and trim parts of adapters or primers. Any sequence with terminal consecutive N’s or more than 1 consecutive internal N was removed. After trimming, only sequences with exact matches to the barcodes and with a length, after barcode trimming, longer than 12 nucleotides were kept and stored in an independent file per sample.

High-throughput Illumina sequencing can be limited by the capacity to accurately map short reads to reference sequenced genomes. In order to increase the precision of mapping reads, the reads were initially classified by sample and assembled to generate contigs that could be more easily mapped to the reference *M. tuberculosis* genomes. This strategy allowed us to identify genomic locations using contigs of up to 300 nucleotides rather than 35-nucleotide reads. Sequences from each sample were assembled using Velvet [48], parameters were adjusted for a seed of 17 bases and short pair ends. During the assembly the minimum coverage cutoff was 4, the expected insert length was 400 and the minimum contig length was 45. After the assembly, all adapter reads were mapped back to the contigs, and the corresponding primer read was retrieved. Since the primer read was trimmed for the IS specific primer sequence, all the primer reads should begin with the target sequence at the exact position of the insertion site. For each contig, all the primer reads were aligned using clustalW to generate a consensus sequence. If the consensus had less than 85% conservation and coverage less than 10, the contig was dissolved and the reads sent to a leftover file.

For the remaining contigs, the consensus of the alignment and the contig sequence were used as queries against the 6 complete and annotated *M. tuberculosis* genomes (Genbank accession numbers: NC_000962.2, NC_002755.2, NC_002945.3, NC_008769.1, NC_009525.1, NC_009565.1). All hits were retrieved and organized by genome according to the detail level of annotation. If the contig and the consensus hit the same genome in opposite strands and with a maximum distance between the hits of 600 bp (since the fragments sequenced ranged between 200–500 bp), the hit was reported in a table as a “specific” hit where the coverage, genome, position and orientation of the hit were reported. Otherwise, the contig was dissolved and the long reads sent to the leftover file. Leftover reads were mapped individually to all the sequenced reference genomes. All hits within 10% of the score of the best hit were kept for each read. Each region in the reference genomes longer than 25 bp with a minimum coverage of 9 reads was reported. In this case, since there is no evidence for the exact insertion site location, the fragments are labeled as “approximate”. Both reports were combined and a table for each strain was generated with the coverage, position, strand, genome and the annotation for the position.

Since both ends of each insertion element were ideally amplified and sequenced, we further parsed the data in order to search for segments located in close proximity but in opposite directions. This parsing generated 5 categories of results based on the confidence of the mapping. C0 corresponded to IS located in the CRISPR element of the genome known as the direct repeat (DR) region, which due to tandem 36 bp repeats needed special parsing for accurate mapping. C1, which has the highest confidence, consisting of a pair of “specific” fragments each corresponding to an end of the IS element. C2 consisted of a pair of fragments, one” specific” and one “approximate” in close proximity and opposite directions. C3 was formed by two” approximate” fragments in close proximity and opposite directions. C4 indicated that only one “specific” fragment could be mapped. C5 include those hits where only an “approximate” fragment was mapped. These last 2 categories had the highest probability of being false positives but they were kept since they could also represent regions where one of the flanking sides of the IS element is absent from all 5 reference genomes due to strains specific insertions or deletions.

### Coverage cutoff determination

To see how well our sequence reads were able to detect IS insertion sites we looked at the correlation between expected (based on RFLP data) and observed sites (our IS-seq protocol). The relationship between the coverage obtained using IS-seq (reads per RFLP band) and the difference between observed and expected sites showed that our method was prone to identify fewer insertion sites than number of bands identified by RFLP when the coverage was below 100 reads per RFLP band, hence decreasing the sensitivity (Additional file 3: Figure S 2). For this reason, 60 strains with low coverage were discarded from further analysis. At high coverage the curve leveled off with an average of 4 observed sites above the number of RFLP-detected bands, reflecting again that RFLP analysis underestimates the real copy number of the IS*6110* element. This also implies that there will be no increase of false positives with higher depth of sequencing.

### Definition of unique insertion sites

In order to create matrices and compare insertion sites among samples it was necessary to determine which insertion sites were conserved among strains. For this, the different observed sites were evaluated based on the estimated precision. Initially a seed matrix was generated with all C1 sites that constitute the most accurate sites identified. Sites classified as C2, C3 or C4 were then compared to the seed, if they fell within 30 bp of a C1 site, both were collapsed; otherwise a new unique site was created. C0, which represent insertions on the DR region, were checked manually for determination of independent insertion sites. Within each locus and for insertion sites identified in homologous genes of different reference genomes, a manual curation was done to collapse a site when each terminus of the insertion mapped with high scores to different genomes (i.e. H37Rv and CDC1551). Unique sites with more than 20 sequences were checked manually for consistency and to verify that it was not composed of more than one unique site within the parameter of 30 bp used above.

### Data analysis and statistics

Since complete genome sequences of all the strains under study are unavailable, and thus estimation of true negatives is unfeasible, we calculated the sensitivity and positive predictive values (PPV) for validation analysis using the following formulas:

(1) $$Sensitivity=\frac{True\;Positives}{True\;Positives+False\;Negatives}$$

(2) $$PPV=\frac{True\;Positives}{True\;Positives+False\;Positives}$$

### Rarefaction analysis

We assumed that each insertion observed appeared either independently or that all the insertions in a given position of the genome appeared only once in a common ancestor. Although both assumptions are unlikely, they nevertheless give the lowest and highest maximum number of insertion events that can be measured. To rarefy this number of insertion events respective to the number of genes affected, random sub-sampling of the distribution of insertions was performed continually from a total of 10 insertions to the total number of insertions available. A step of 20 insertions was chosen and 10 replicates per step were performed. The data was plotted in MATLAB and a non-linear fit to an exponential decay function was performed using the formula:

(3) $$Y=k\left(1-e^{-\alpha x}\right)$$

### Distribution of IS sequences

To examine the bias in genomic distribution of IS sequences in the *M. tuberculosis* genome, the number of independent insertions observed in non-overlapping windows of 2 and 50 Kb were determined. Given the total number of independent insertions and the length of the genome, an average number of insertions in a 2 or 50 Kb window can be determined by,

(4) $$Expected=\frac{No\;Independent\;Insertions}{Genome\;Length}\times Window\;size$$

We tested the probability that the observed number of insertions in a given window followed an expected Poisson distribution. Bonferroni correction of the threshold was performed to assess the significance of the hit.

### Functional category representation

COG categories and gene lengths were retrieved from the NCBI entry for each reference genome (accessed on August 1, 2011). Genes with independent insertions were assigned to the different categories, filtering out those belonging to the PE/PPE family or those corresponding to IS*6110* elements. The Fisher exact test was performed on each category to estimate the probability of obtaining by chance that number or more insertions in a particular category, given the length of the genes. Bonferroni correction for the threshold was performed. The genes involved in virulence, detoxification and adaptation were retrieved from http://tuberculist.epfl.ch/index.html (Accessed on July 26, 2011).

### Variation of information and conditional information

To determine the relatedness among the different isolates given by IS*6110* insertion patterns, we applied mutual information measurements. In order to generate a metric that will allow us to build hierarchical clustering trees, we used the derived metric known as Variation of Information where the distance between any two samples d(X,Y) is given by: Equation 1:

(5) $$d\left(X,Y\right)=\frac{H\left(X\right)+H\left(Y\right)-2I\left(X,Y\right)}{H\left(X,Y\right)}$$

where I(X,Y), or the mutual information between X and Y, is defined as Equation 2:

(6) $$I\left(X,Y\right)=\sum_{y\in Y}\sum_{x\in X}p\left(x,y\right)\log\left(\frac{p\left(x,y\right)}{p\left(x\right)p\left(y\right)}\right)$$

and H(X) is defined as Equation 3:

(7) $$H\left(X\right)=\sum_{x\in X}p\left(x\right)\log\left(p\left(x\right)\right)$$

Once we determined that the IS*6110* insertion sites were highly associated with strain classifications we used Mutual Information (Equation 2) to determine the association between a given insertion in a loci and a specific family. Mutual Information values can be used to determine approximate p-values for the association as follows:

(8) $$pvalue=2^{-I\left(X,Y\right)\times N\left(X\right)}$$

where N(X) is the number of elements of X.

We used Conditional Mutual Information, which allows us to compensate for the correlation between insertion sites and phylogeny, to test for correlation between biological traits (X) and the presence or absence of a particular insertion site (Y) given that the strain belongs to a phylogenetic family (Z), using the following formula:

(9) $$I(X;Y|Z)=\sum_{z\in Zy}\sum_{\in Yx}\sum_{\in X}p_{X,Y,Z}\left(x,y,z\right)\times \log\left(\frac{P_{Z}\left(z\right)P_{X,Y,Z}\left(x,y,z\right)}{P_{X,Z}\left(x,z\right)P_{Y,Z}\left(y,z\right)}\right)$$

### Family prediction of sequenced available genomes

Fasta files were downloaded for all available *M. tuberculosis* genomes present in the PATRIC database [49] on September 25 2010: *M. tuberculosis* '98-R604 INH-RIF-EM', *M. tuberculosis* 02_1987, *M. tuberculosis* 210, *M. tuberculosis* 94_M4241A, *M. tuberculosis* C, *M. tuberculosis* CDC1551, *M. tuberculosis* CPHL_A, *M. tuberculosis* EAS054, *M. tuberculosis* F11, *M. tuberculosis* GM 1503, *M. tuberculosis* H37Ra, *M. tuberculosis* H37Rv, *M. tuberculosis* K85, *M. tuberculosis* KZN 1435, *M. tuberculosis* KZN 4207, *M. tuberculosis* KZN 605, *M. tuberculosis* KZN R506, *M. tuberculosis* KZN V2475, *M. tuberculosis* T17, *M. tuberculosis* T46, *M. tuberculosis* T85, *M. tuberculosis* T92, *M. tuberculosis* str. Haarlem. IS *6110* elements were identified in each genome using blast with an e-value threshold < 1e-30; 200 bp of the up-stream and down-stream flanking sequences were extracted for each insertion identified. The positions of these insertions were identified running blast of the flanking sequences against the H37Rv and CDC1551 genomes (NCBI). The list of insertions and positions was compared against the family-specific markers identified and were classified accordingly. A subset of these strains has been used recently and classified using SNPs (see Additional file 1: Table S 7).

## Competing interests

The author(s) declare that they have no competing interests.

## Authors’ contributions

Conceived and designed the experiments: AR, AS, KEV, MMZ, RDM, PDP. Performed the experiments: AR, AS, AC, IH. Analyzed the data: AR, AS, SS, CM, MJG, VR, LL, JR, MMZ, RDM, PDP. Wrote the paper: AR, AS, AC, IH,SS, CM, MJG, VR, JR, MMZ, RDM, PDP. All authors read and approved the final manuscript.

## Supplementary Material

Additional file 1 **Table S1.** RFLP, barcode and sequencing information for strains **. Table S2** Loci with identified IS *6110* insertions. **Table S3** 2kb and 50Kb window analysis for IS distribution **. Table S4** Loci with insertion sites in multiple strains **. Table S5** Characteristic insertion sites associated with specific *M. tuberculosis* lineages. **Table S6** Classification of strains based on spoligotyping, RFLP and IS-seq **. Table S7** Classification of sequenced *M. tuberculosis* genomes using IS-seq data **. Table S8** Association between characteristic IS *6110* and biological traits by mutual information. **Table S9** Barcodes used in primers and adapters. **Table S10** Primers used for insertion site validation. Click here for file

Additional file 2 **Figure S1.** Distribution of Barcodes in Sequenced Samples. Out of approximately 14 million reads (2 x 35/60 bp), 13,243,263 contained a barcode from the adapter (A) and 8,313,986 contained the barcode and the IS *6110* specific primer (B). Different colors represent different barcodes used. The barcode in the IS *6110* specific primer was more evenly distributed (332,559 ± 84,930 reads per barcode) than the barcode in the adapter (551,803 ± 322, 632 reads per RFLP band). In the latter case the outlier barcodes corresponded to CCGG and CACGA that can potentially generate a hairpin with the adapter sequence, thus hampering the ligation reaction. Click here for file

Additional file 3 **Figure S2.** - Detection of Insertion Sites Using IS-seq. The difference between observed (Number of insertions determined by IS-seq) and expected (Number of RFLP bands) sites is plotted against the coverage (reads per strain) obtained with IS-seq. Error bars indicate standard deviation. Red line shows the limit at which lower coverage resulted in reduced specificity of detection. Click here for file

Additional file 4 **Figure S3.** Rarefaction Analysis of Insertion Sites. Random subsampling of the number of insertion sites were performed at different depths and plotted against the number of genes interrupted. Plots for all the insertions observed (green) or for unique insertions (blue) were performed. Non-linear fits for an exponential decay function were estimated (orange and black lines). Coefficient of determination (R^2^) for each regression is shown as well as the estimator for the K parameter, where K represents the maximum theoretical limit for each regression model, which corresponds to the lower and higher limits of the maximum number of genes, predicted to be susceptible to IS*6110* in vivo. Click here for file
