# Supplementary material for: IS-seq: a novel high throughput survey of in vivo IS6110 transposition in multiple Mycobacterium tuberculosis genomes
Source: BMC Genomics. 2012 Jun 15;13:249. doi: 10.1186/1471-2164-13-249 (PMC3443423; doi:10.1186/1471-2164-13-249)
Supplement: Additional file 2 — Figure S1. Distribution of Barcodes in Sequenced Samples. Out of approximately 14 million reads (2 x 35/60 bp), 13,243,263 contained a barcode from the adapter (A) and 8,313,986 contained the barcode and the IS 6110 specific primer (B). Different colors represent different barcodes used. The barcode in the IS 6110 specific primer was more evenly distributed (332,559 ± 84,930 reads per barcode) than the barcode in the adapter (551,803 ± 322, 632 reads per RFLP band). In the latter case the outlier barcodes corresponded to CCGG and CACGA that can potentially generate a hairpin with the adapter sequence, thus hampering the ligation reaction. [file 1471-2164-13-249-S2.pdf]

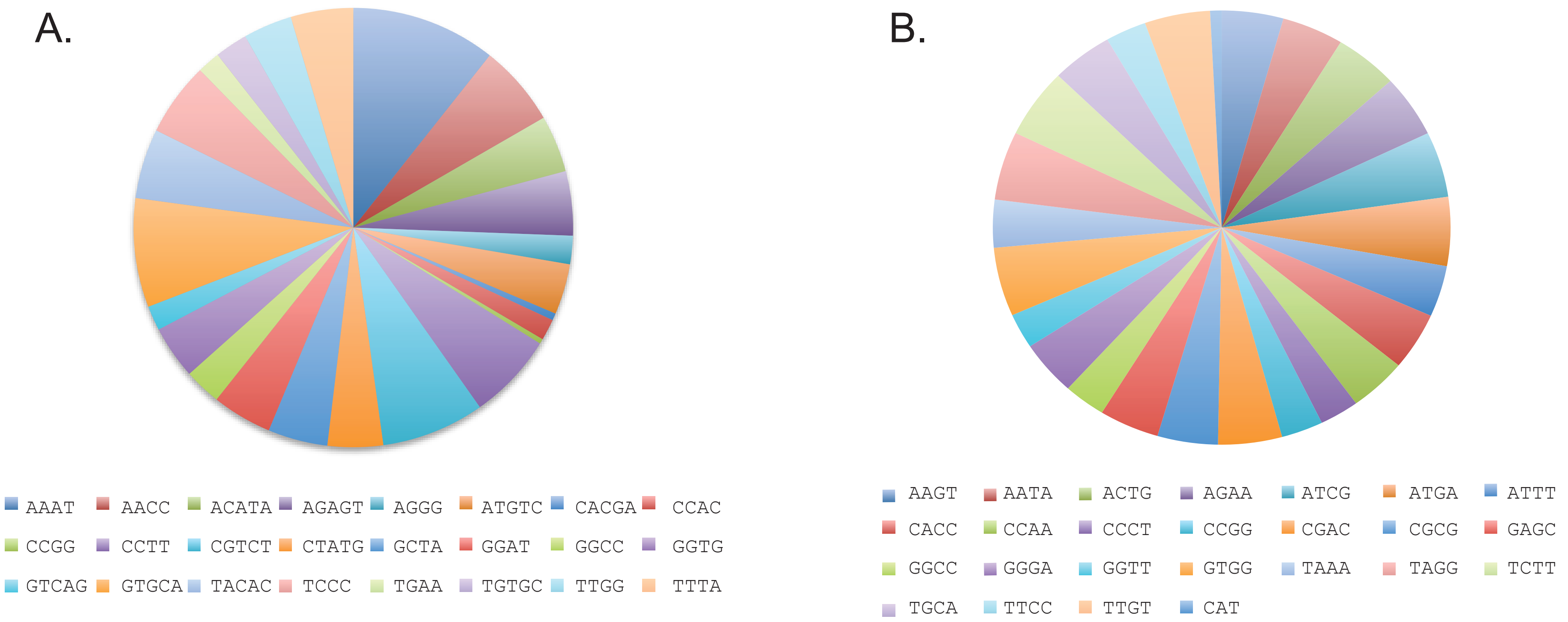

A.
AAAT
AACC
ACATA
AGAGT
AGGG
ATGTC
CACGA
CCAC
CCGG
CCTT
CGTCT
CTATG
GCTA
GGAT
GGCC
GGTG
GTCAG
GTGCA
TACAC
TCCC
TGAA
TGTGC
TTGG
TTTA
B.
AAGT
AATA
ACTG
AGAA
ATCG
ATGA
ATTT
CACC
CCAA
CCCT
CCGG
CGAC
CGCG
GAGC
GGCC
GGGA
GGTT
GTGG
TAAA
TAGG
TCTT
TGCA
TTCC
TTGT
CAT
