# Supplementary figures and images for: IS-seq: a novel high throughput survey of in vivo IS6110 transposition in multiple Mycobacterium tuberculosis genomes
Source: BMC Genomics. 2012 Jun 15;13:249. doi: 10.1186/1471-2164-13-249 (PMC3443423; doi:10.1186/1471-2164-13-249)

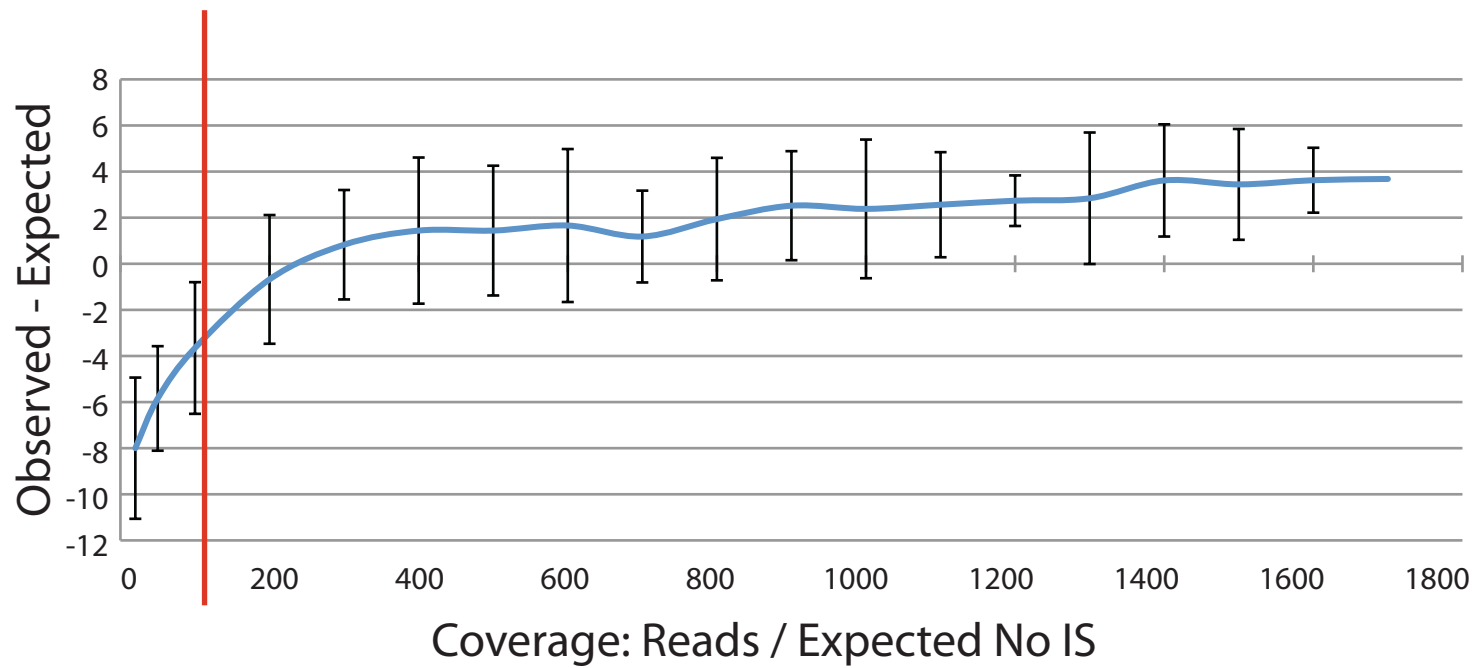

Supplement: Additional file 3 — Figure S2. - Detection of Insertion Sites Using IS-seq. The difference between observed (Number of insertions determined by IS-seq) and expected (Number of RFLP bands) sites is plotted against the coverage (reads per strain) obtained with IS-seq. Error bars indicate standard deviation. Red line shows the limit at which lower coverage resulted in reduced specificity of detection. [file 1471-2164-13-249-S3.pdf]

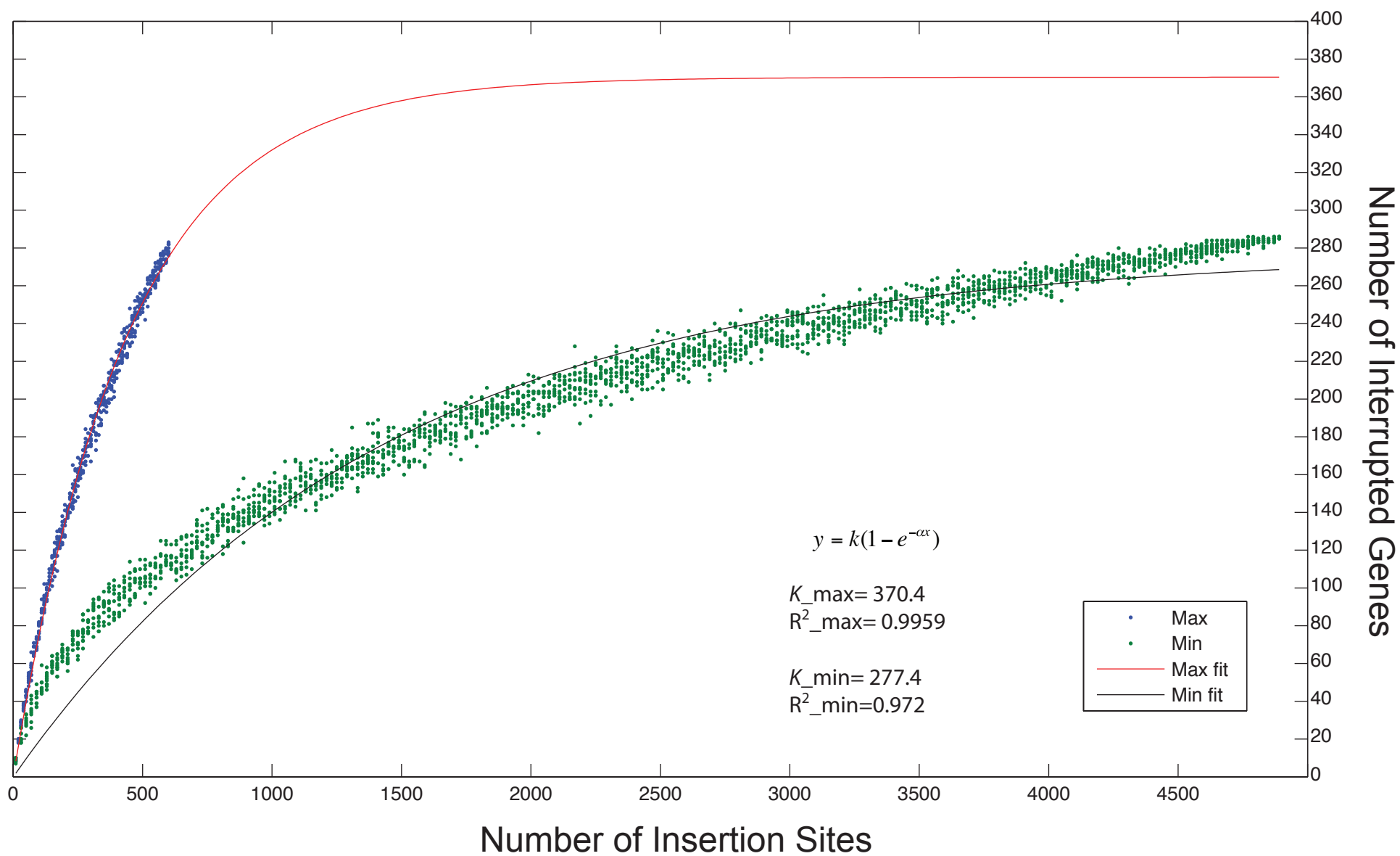

Supplement: Additional file 4 — Figure S3. Rarefaction Analysis of Insertion Sites. Random subsampling of the number of insertion sites were performed at different depths and plotted against the number of genes interrupted. Plots for all the insertions observed (green) or for unique insertions (blue) were performed. Non-linear fits for an exponential decay function were estimated (orange and black lines). Coefficient of determination (R2) for each regression is shown as well as the estimator for the K parameter, where K represents the maximum theoretical limit for each regression model, which corresponds to the lower and higher limits of the maximum number of genes, predicted to be susceptible to IS6110 in vivo. [file 1471-2164-13-249-S4.pdf]
